# Ant Colony Optimization Approaches to Clustering of Lung Nodules from CT Images

**DOI:** 10.1155/2014/572494

**Published:** 2014-11-26

**Authors:** Ravichandran C. Gopalakrishnan, Veerakumar Kuppusamy

**Affiliations:** ^1^SCAD Institute of Technology, Palladam, Coimbatore 641664, India; ^2^Department of ECE, RVS College of Engineering and Technology, Dindigul 624005, India

## Abstract

Lung cancer is becoming a threat to mankind. Applying machine learning algorithms for detection and segmentation of irregular shaped lung nodules remains a remarkable milestone in CT scan image analysis research. In this paper, we apply ACO algorithm for lung nodule detection. We have compared the performance against three other algorithms, namely, Otsu algorithm, watershed algorithm, and global region based segmentation. In addition, we suggest a novel approach which involves variations of ACO, namely, refined ACO, logical ACO, and variant ACO. Variant ACO shows better reduction in false positives. In addition we propose black circular neighborhood approach to detect nodule centers from the edge detected image. Genetic algorithm based clustering is performed to cluster the nodules based on intensity, shape, and size. The performance of the overall approach is compared with hierarchical clustering to establish the improvisation in the proposed approach.

## 1. Introduction

Lung cancer is one of the most common types of cancers in the world. The impact of lung cancer differs based on the stages of its occurrence. Lung cancer is generally detected based on the presence of lung nodules. Lung nodules are very common and can be seen in 1 out of 500 scans. They are small masses of tissue in the lung. Cancerous nodules are referred to as being malignant. Pleural nodules are tiny parts of tissues within the lung. Lung nodules are in various shapes, spherical, oval, and hemisphere.

Computer tomography (CT) scan images are widely used to locate the lung nodules. CT screening enables detection of lung cancer at early stages and helps continuous monitoring at later stages. Computer aided detection (CAD) helps radiologists to quickly and accurately estimate the size and growth percentage of the nodules. During successive monitoring CAD systems help to detect nodules growth with the help of previous screening and presence of new nodules at present screening.

Lungs nodules are generally spherical in shape. Malignant nodules are larger when compared to benign nodules. A 5-year prospective experience reveals that the diameter of lung cancer will be 5–50 mm with an average of 14.4 mm [1]. Lesions greater than 3 cm are usually classified as being malignant and smaller lesions are classified as being benign. Nodules with larger diameter should be given greater care when compared to small diameter nodules.

Lung nodule detection initially involves edge detection from the lung images. Basically there are two approaches, namely, gradient and Laplacian. In gradient approach, edge is detected from the first derivative maximum and minimum values and, in Laplacian approach, we use the second derivative zero crossing of the image and variant ACO algorithm [2], which uses the result of another algorithm as the input. There are three basic operators which form the basis for edge detection. They are Sobel operator, which detects edges using 3 × 3 convolution matrixes, Robert cross operator, which detects edges using 2 × 2 convolution matrixes, and Prewitt operator, which is similar to Sobel operator and is used to detect vertical and horizontal edges in image.

Lung nodule detection starts with segmenting the lung region from the input image. The initial step segments the lung region separately where the nodules are supposed to be located. Then the segmented region is processed for nodule enhancement. In this work we discuss the application of ACO algorithms for pulmonary nodule detection and suggest some variations in the ant colony optimization algorithm to arrive at better results. Other algorithms, namely, Otsu algorithm, watershed algorithm, and global region based segmentation algorithm, are compared with the performance of ACO. In addition, we propose three levels of improvements to ant colony optimization algorithm. The first level is extending the number of iterations based on its performance criteria, thereby refining the output of normal ACO. The second is that the refined outputs are XORed to make the output more logical. The third level alteration involves giving the output of another algorithm, say Otsu, as input to ACO algorithm.

Later, candidate nodule selection is done to identify the lung nodules. Since large number of false positives (FPs) is included in the candidate nodule selection stage, we need to go for 3-stage FP reduction process. Though these three steps are included in many of the existing systems, in this work, we propose a different approach by initially choosing an edge detection algorithm for segmenting. From the edge detected output, we propose a new methodology for candidate nodule selection and FP reduction.

Computer aided detection systems are widely used to detect and diagnose numerous diseases and are a prime area of research in medical imaging. Most common anatomical regions covered under CAD are lung, chest, breast, and brain. Summers, [3] says that CT or MRI scan images are popularly used as input for such detection systems. Doi, [4] clarifies the fact that the popular and significant applications include locating clustered microcalcifications in mammograms and pulmonary nodule detection for lung cancer. Most commonly knowledge based approaches provide better results in CAD applications. Commonly used classifiers are rule-based, k-NN, ANN, decision trees, Naïve Bayes, LDA, and SVM. Out of these, Korfiatis et al. [5] present rule-based classifiers which are simple to implement and can be integrated with other classifiers to produce better results. However, selection of disease identification threshold has to be done manually. CAD is popularly applied for pulmonary nodule detection using morphological features [6]. *k*-nearest neighborhood approach given by Chen et al. [7] and Zrimec and Wong [8] provides consistent classification results. But this method has the problem of identifying the correct “*k*” value which shows improved accuracy with larger datasets.

EM algorithms are well applied for lung nodule detection and segmentation for the earliest detection of lung cancer nodules [9]. Lung volume is segmented using adaptive border marching algorithm. Region growing approach is used for detection of candidate lung nodules. Further, features including intensity and geometric features were extracted. The nodules were then classified using fuzzy min-max neural network classifier enhanced by K-means clustering [10].

To fill the gap of semantic description of lung nodules collected from patients, Lung Image Database Consortium (LIDC) database presents annotated pulmonary CT scans, which is used for various image query and retrieval purposes [11]. However, this database is not widely used for lung nodule detection research until now. Low dose CT scan images are also equally used for lung nodule detection [12]. Multiresolution feature analysis combined with template matching resulted in improved lung nodule detection from low dose CT images. Appearance based template matching [13] is also introduced. Genetic approaches [14, 15] to template matching show promising improvement in performance. Feature based template matching involving texture and shape based features is also attempted [16].

Artificial neural networks proposed by Pun and Lee, [17] are yet another remarkable milestone in medical imaging applications. The completeness and consistency of performance provided by ANN based applications are due to the ability of system to learn itself completely from the input-output combinations independent of domain specific issues. The reason for less popularity of ANN is that this required heavy dose of training to guarantee error-free and consistent learning, which is not always possible. Alternatively, decision tree based learning that attempted to grab the attention of ANN researchers in medical imaging is mentioned by Kauczor et al. [18]. Unlike ANNs, decision tree learning has low computational complexity and less training. However, overfitting was the potential drawback in decision trees. Neither ANN nor decision trees were able to solve the problem of CAD of diseases. Then there appeared the optimal approach called Naïve Bayes classification, where the problems of both ANN and decision trees were ruled out.

Yet another scheme for classification much simpler than Naïve Bayes is known as LDA [19]. This approach was analytically simple and computationally less expensive. LDA demands selection of appropriate feature sets of input images for better classification results. The most improved optimal solution for classification is support vector machines. Though this approach has very high algorithmic complexity, the results of SVM training and classification were close to human inferences. Adaptive distance based threshold is also used to identify lung nodules. Using this, Fisher Linear Discriminant (FLD) classifier is used. The dataset created in the process was by the Standard Digital Image Database Project Team of the Scientific Committee of the Japanese Society of Radiological Technology (JRST). Cellular learning automata are also applied for automatic detection of lung cancer [20].

CAD of pulmonary nodules works with two phases: pulmonary nodule detection and nodule pattern and nodule shape identification. Nodules are generally irregularly round and opaque. They may be solid, nonsolid, or partly solid and exist with less than 3 cm diameters [21]. Nodular patterns are similar to nodules but vary from 2 to 10 mm in diameter and are generally widely spread over the lung regions. In addition, micronodules are less than 3 mm in diameter. A disease affected lung region shall contain one or many or combination of nodules and nodular patterns. Therefore, computer aided detection of nodules and nodular patterns includes analysis of shape based features in CT images. Studies made by Böröczky et al., [22] reveal that size, volume, area, diameter, 2D and 3D dimensions, circularity, solidarity, thickness, top-hat filtering, mean curvature, shape index, Gaussian curvature, sphericity, surface smoothness, shape irregularity, roundness, center of mass, compactness, inertia matrix, and surface curvature are the useful and effective features for pulmonary lung nodule detection and nodule pattern analysis [7, 22–29]. In addition, histogram based approaches and gradience information are also used in detection of small cavities in radiograph images. Several studies by Shen et al. [30] include combination of shape based and texture based methods for pulmonary lung nodule detection.

Content based image retrieval (CBIR) algorithms are also applied for lung nodule detection and classification [31]. In yet another interesting research [32] computer-derived weak segmentation algorithm is proposed which is later used in classification algorithms for cancerous nodule prediction.

Ant colony algorithms are widely applied in wireless networks, especially in routing and load balancing [33]. Very little research focus is put on applying ACO algorithms to medical imaging [34]. These include application of ACO to lymph node classification [35], brain tumour segmentation [36, 37], hippocampus segmentation [38], prostate cancer classification [25], diabetes diagnosis [39], retinal vessel detection [40], ovarian cancer detection [41], and heart ventricle segmentation [42]. Significantly visible research has been performed over applying ACO for microcalcification detection of mammograms by Lochanambal and Karnan, [43]. A remarkable milestone in CAD of lung nodules is reported by van Ginneken et al., [44]. This work combines the output of various algorithms for automated pulmonary nodule detection of CT images and obtains better improvement in performance. In this context, this paper experiments ant colony based approaches for automatic lung nodule detection and combines the output of various ACO and non-ACO algorithms to obtain remarkable performance improvements.

Generally nodule shape and size are considered as the features for nodule detection systems. The lung nodule diameter helps to take further actions for classifying the nodules [45]. When lung nodules are less than 7 mm in diameter, follow-up diagnostic is needed. The nodules which are 8–20 mm in diameter need immediate diagnostic CT. And for nodules greater than 20 mm in diameter CT, PET, or biopsy is made and removal is done. Other than shape and size features many other features are also used to identify the nodules. Cavouras et al. [46] proposed solitary pulmonary nodule discriminating (SPND) system consisting of two phases where the first phase uses 20 features with SPN matrix and the second phase uses Least Squares Distance Measure (LSDM) classifier algorithm upon the 20 features. The features are based on the textual features CT density matrix of SPN, SPN density histogram, and cooccurrence matrix of SPN. The system differentiates benign and malignant nodules from CT images. If additional features such as nodules contour, size, and CT density measurements are obtained, the system may produce higher accuracy rate. In this context, we have additionally selected the intensity of lung images to improve the overall efficiency.

Shape based detection of lung nodules is proposed in [47]. Popular classifiers are widely applied for classifying benign nodules from malignant ones. Feature based nodule detection approaches have concluded that laws and wavelet features contribute more to image classification. Using texture features for detection and classification may result in improved classification accuracy [48].

An existing system uses the contextual information of the image [49] to identify the lung nodules. Context is described by means of high-level features based on the spatial relation between static contextual features and dynamic contextual features. Initially local features are extracted and, in the second phase, context based feature extraction was made. Both local classifiers and contextual classifiers were used to classify identifications in a CT scan image. Contextual classification includes hard exudates binary classification, drusen binary classification, cotton wool spots binary classification, and nonlesion binary classification. When conceptual information is used, the CAD system is similar to human's observation.

Most lung cancer detection methods involve segmentation in their first step [50]. Segmentation of CT image involves the segmentation of lung, the airways and vessels portion, and finally the lobar segmentation. Lung cancer detection is done by detection of pulmonary nodules, characterization of pulmonary nodules, and nodular size measurement. The detection of nodules is done by primarily selecting the candidates using one of the following methods, namely, multiple gray-level thresholding, mathematical morphology, and connected component analysis of thresholded images. False positives are reduced in the following process along with the classification. Fast lung nodule detection is proposed [51] which undergoes the normal method for lung nodule detection of lung segmentation, nodule enhancement, and finally false positive reduction. For nodule enhancement, cylindrical shape filter is used and Support Vector Machine (SVM) with seven types of parameters is applied to reduce the false positives. It fails to detect nodules that are close to or sticking to the lung walls or blood vessels. However, in this work, we assume the whole image for input purposes, and hence this issue is not applicable.

Almost every nodule detection research has attempted to improvise their detection accuracy and reduce false positives. The crux lies in the variation of appearance of potential lung nodules and also in the imbalanced distribution of nodules in the dataset. This is overcome by extending random subspace method, thereby injecting more diversity in the dataset [52]. Template based nodule models are applied for nodule detection and segmentation [53]. To reduce false positives, consecutive CT slices are ANDed to provide a more logical approach [54]. Simultaneous optimization of SVM classifier is also attempted [55]. However, in such approaches, misclassification cost is enormous. In addition, selection of optimal features and kernel function remains alternate issues [56].

Murphy et al. [57] presented an approach which uses the local features like shape index and curvature. Here two successive k-nearest-neighbors (KNNs) are applied to reduce the false positives. Using the shape index and curvedness, seed points within the lungs are detected by thresholding. A region growing algorithm is applied to form clusters which are then merged and location adjustment is made. Upon the clusters, KNN algorithm is applied. To reduce the false positives, again KNN clustering algorithm is applied and nodules are localized. Since conventional region growing required human intervention in selecting the seed points, threat point identification is proposed [58] for segmenting the suspicious regions.

To reduce the detection of unwanted lung nodules, a vowel-based neural approach is also applied [59]. Here, spherical shape objects are detected and neural approach is used to detect the lung nodules from the CT images. The lung portion is segmented and nodule candidates are selected. This approach identifies the region of interest using ROI hunter algorithm. It eliminates false positives using vowel-based approach. The nodules listed are classified using a neural classifier and tagged as nodule if voxels percentage is higher than a certain threshold. It is helpful for finding nodules whose diameter is greater than 5 mm only. An iris filter is used to discriminate the false positives from the nodules [60]. Regions were characterized by iris filter output and morphological features are extracted from CT images. To reduce the false positives iris filter is used with the Linear Discriminant Analysis (LDA). As the name suggests, the iris filter can be used as second reader for radiologists.

A study has been made by applying many systems over the same set of images [44]. The dataset selected was ANODE09. Six algorithms, namely, Fujitalab, region growing volume plateau, Channeler ant model, voxel-based neural approach, ISI-CAD, and Philips Lung Nodule CAD, are applied and their performances were compared. Many clustering algorithms have been proposed of which the most popular algorithms are *k*-means, spectral graph clustering, suffix tree based clustering, genetic algorithm based clustering, and hierarchical clustering. In this work, we used genetic algorithm for clustering. It greatly helps to find the exact cluster centers. The detection of 80% nodules suggests that blending of algorithms gives a right path for new inventions.

This paper elaborates on a new concept for identifying malignant lung nodules from CT scan images. The idea proposed helps to identify malignant nodules by classifying them with their intensity, shape, and size. The CT images are used as input because we use intensity as one of the features in our approach. For edge detection we apply variant ant colony optimization (variant ACO) algorithm [2]. Variant ACO is chosen because it helps to reduce the number of false positives at the earlier stage. We use intensity, shape, and size as our features because lung nodules are classified according to these features as being malignant and benign. We concentrate on detecting malignant nodules because malignant nodules are cancerous in nature. The final evaluation results show that we achieve expected output by highly reducing the number of false positives.

## 2. Materials and Methods

### 2.1. Proposed Idea: ACO Based Detection and Clustering

Initially the lung CT image is given as the input to the edge detection algorithms. Various edge detection algorithms, namely, Otsu, watershed, global region based segmentation, ant colony optimization (ACO), variant ACO, refined ACO, and logical ACO, are applied over the lung CT image. From the observation made, variant ACO gives better performance when compared with other algorithms. Hence variant ACO is used to detect the lung CT image edges at the initial steps. Based on the edge pixels, the lung nodule pixels are identified using the black circular neighborhood algorithm [2]. The nodules thus identified are clustered using genetic algorithm based on shape, size, and intensity of the nodules.

Edges are changes in intensity of the image. In this section, we present four existing algorithms for edge detection. Since lung nodule detection fundamentally involves edge detection, we use edge detection algorithms as the primary step of lung nodule detection. The detailed working of the algorithms is presented below.

#### 2.1.1. Otsu Algorithm

**Figure 1:**
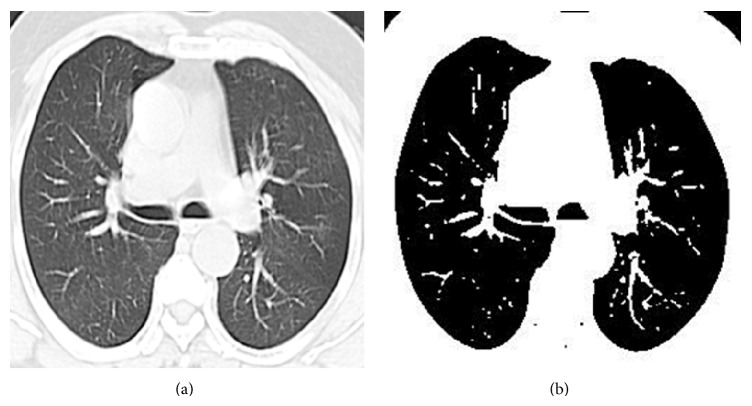
(a) Input lung CT image. (b) Otsu output image.

Otsu uses gray scale images for its image processing steps. Hence we take a gray scale lung CT images for further processing. In this method it searches for the pixels with all possible threshold values and finds the spread of pixels in each threshold range. It involves finding the pixels that fall under foreground and background. The edge is detected when the sum of foreground and background spread is the maximum. The mean weight and variance are calculated. Then within class variance is calculated whose value is used to detect the edge (refer to Algorithm 1). Figures 1(a) and 1(b) show the input-output of Otsu edge detection.

#### 2.1.2. Watershed Algorithm

**Figure 2:**
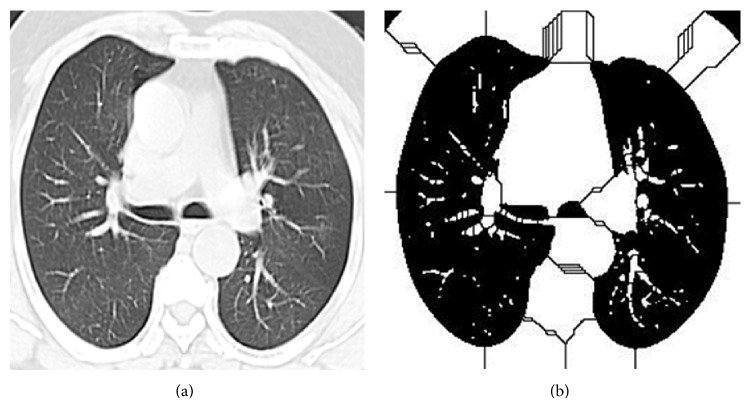
(a) Input lung CT image. (b) Watershed output image.

In grey scale images, different grey levels indicate the edges. Watershed algorithm basically sees the image as topographic relief. The basic idea behind this is construction of dams. The catchment areas refer to the objects we are trying to segment; here the catchment areas are the lung nodules. As the water level increases, dams are constructed to protect ourselves. When the water level reaches the highest peak construction stops. In the same way, we start from the watershed pixels and grow iteratively. When the edge detected reaches the maximum level, the process stops and gives the required edges. The detailed procedure is given in Algorithm 2.  Figure 2(b) represents the edge detection of watershed algorithm for Figure 2(a).

#### 2.1.3. Global Region Based Segmentation Algorithm

**Figure 3:**
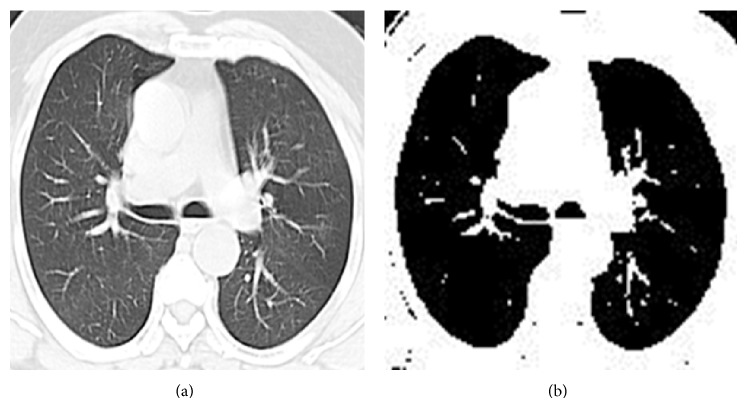
(a) Input lung CT image. (b) Global region based segmentation output image.

The lung fields are segmented in CT image using a region growing algorithm. The algorithm is based on the selection of pixel; the pixel can be selected either by giving (*x*, *y*) coordinate or by clicking a pixel from the CT image. After selecting the pixel, the regions associated with this pixel based on connectivity and gray scale difference were formed by using the region mean. Through this method the given CT images were segmented and lung nodule edges are detected. The procedure of global region based segmentation is given in Algorithm 3. For Figure 3(a), the input image, the edge detection by global region based segmentation is shown in Figure 3(b).

#### 2.1.4. Ant Colony Optimization Algorithm

**Figure 4:**
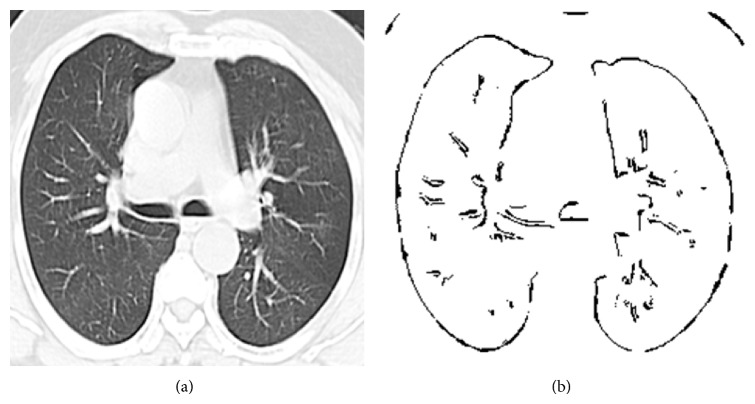
(a) Input lung CT image. (b) Ant colony optimization output image.

The basic idea behind this algorithm is the movement of ants. All ants follow the same path with the help of pheromone which will be left by the preceding ants. The succeeding ants make use of this pheromone to find its path. Therefore each ant incrementally constructs a solution to the problem. Likewise, every ant constructs the edge of input image which is done iteratively to obtain the edges of lung nodules. The detailed procedure is given in Algorithm 4. Figures 4(a) and 4(b) present the input and output of ant colony optimization algorithm.

### 2.2. Proposed Novel ACO Approach for Lung Nodule Detection

We propose three different levels of alterations of normal ACO algorithm. Initially we extend the number of iterations for lung nodule detection and then the refined outputs which are XORed to improve its output value and finally tried to give the output of another edge detection algorithm; we have used Otsu algorithm, to be fed as input to the ACO algorithm. Detailed explanations and outputs are given in the following section.

#### 2.2.1. Refined Ant Colony Optimization Algorithm

**Figure 5:**
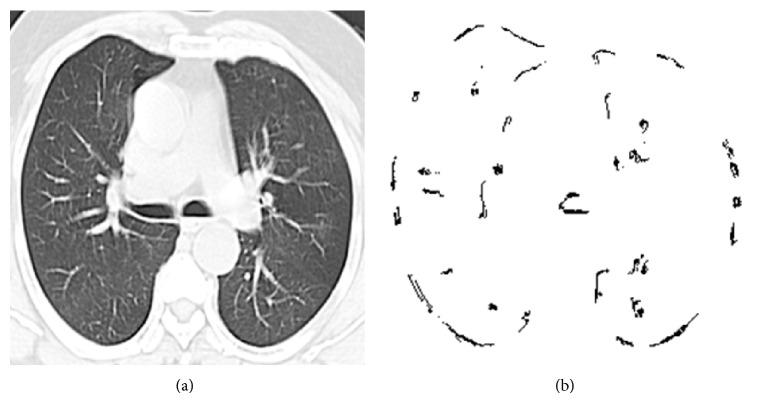
(a) Input lung CT image. (b) Refined ACO final output image.

From the results of normal ant colony optimization algorithm, we notice that some improper edges are detected. To overcome this, the idea of giving the output of previous iteration as the input to the next iteration helps to improve the performance. Since we are refining the iterations we get a better output than the normal Ant Colony Optimization algorithm. This is continued until the differences of subsequent iterations do not alter much. The pseudocode is presented in Algorithm 5.  Figure 5(b) shows the improved edge detection results for the image in Figure 5(a).

#### 2.2.2. Logical Ant Colony Optimization Algorithm

**Figure 6:**
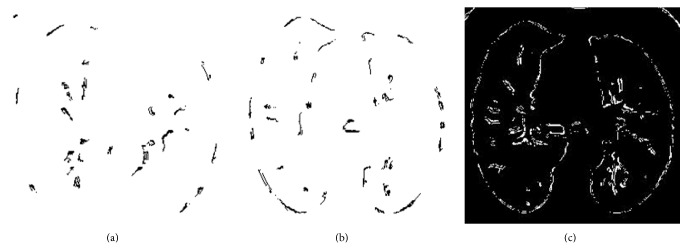
(a), (b) Logical ACO input (refined ACO final and prefinal output images). (c) Logical ACO output image.

From the refined Ant Colony Optimization algorithm's output and normal Ant Colony Optimization algorithm's output, we notice that it detects noises along with the nodules. Hence a logical operation is applied to get even better detection of lung nodules. We get the final iteration output of refined ACO and the previous iteration output of refined ACO algorithm and then apply XOR to it to get the logical ACO output. This shows further reduction of noises in the output image. Figures 6(a) and 6(b) are the input to logical ACO and Figure 6(c) shows the output after applying logical ACO. The detailed pseudocode is given in Algorithm 6.

#### 2.2.3. Variant Ant Colony Optimization Algorithm

**Figure 7:**
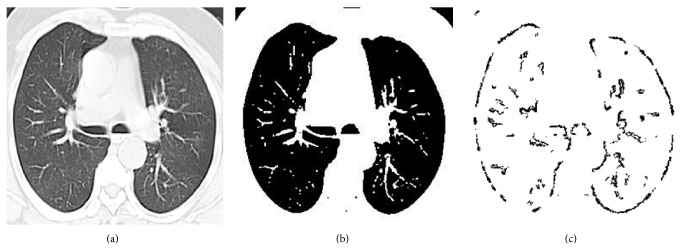
(a) Input CT scan image. (b) Output from Otsu algorithm. (c) Output of variant ACO algorithm.

ACO algorithm is based on the idea of pheromone left by the ants. The ants following the previous ants use the pheromone left by its predecessor ants. ACO algorithm leaves behind a large number of false positives. To reduce the number of false positives, variant ACO algorithm can be used to detect the edges of the image [2]. Variant ACO algorithm developed is an alteration of Ant Colony Optimization algorithm. Variant ACO algorithm works on the output of another edge detected image. The lung CT image is edge detected using the Otsu algorithm (refer to Algorithm 7). Then, the ACO algorithm (refer to Algorithm 7) is applied which calculates the probability for the next stage using the previous stage's decision. By taking different paths, the algorithm chooses the best path which gives the edge of the image. By using variant ACO method, noise in the output is very much reduced (illustration in Figure 7). False positives are reduced at the initial stage itself.

### 2.3. Shape and Size Based Detection of Nodule Positions

Lung nodules (both malignant and benign) are mostly spherical in shape. They can be classified based on size. Cancerous (malignant) nodules are larger when compared to noncancerous (benign) nodules. More care should be given to the nodules whose size is greater than 3 cm [41]. While detecting lung nodules, the size has to be detected along with the shape. A spherical region with the specified diameter gives the nodules present in the given CT image. The edge detected image is given as input to black circular neighborhood algorithm [2]. The assumption is that all the edges are black pixels. The edges here represent the edges of the lungs along with the nodule's edges. As the lung edges are fine lines and nodules are identified in the form of clustered black pixels, we apply black circular neighborhood algorithm. The objective of black circular neighborhood algorithm is to find the center pixel of clustered black pixels. Initially, clustered black pixels are identified using the 4-connected and 8-connected properties of the pixels. This algorithm detects nodules whose diameter is up to 5 units. It detects both spherical and elliptical nodules. From the black pixel clusters, the center pixel is identified to be the center of lung nodules. Using the spherical shape and size we shall spot the nodules in the edge detected image. These nodules are later classified based on their intensity.

### 2.4. Intensity Based Filtering

Nodules are identified by the radiologists with the help of intensity change in the CT image. A whiter region on the image indicates the presence of nodule in lungs. The intensity feature of the nodule is used to identify them. In our earlier work [2] we proposed an intensity based feature extraction algorithm for locating the nodules position over the CT image. The center pixels of the lung nodules are retrieved from the black circular neighborhood algorithm. A circular region is determined around the center pixel of the nodules. The intensity of the nodules within the radius of the circular region is retrieved from the input lung CT image. The sum of intensity of all the pixels inside the circular region is calculated and its average value is found. When the average intensity exceeds the threshold intensity, the specified pixel and the size of the surrounding region are entered in the feature matrix. The output feature matrix consists of average intensity of the region, size, and the shape of the region.

### 2.5. Clustering

#### 2.5.1. Hierarchical Clustering

The feature matrix consisting of size (i.e., area of the identified nodules) and intensity with the age of the patient is fed as input. Agglomerative approach is used to cluster the features in a bottom-up manner. The clustering is done by merging two groups having the smallest dissimilarity measure. Nearest-neighbor linkage is used to find the dissimilarity between cluster points.

#### 2.5.2. Genetic Algorithm Based Clustering

The feature matrix is initially given to the *k*-means algorithm to cluster the nodules and the cluster centers are identified. The cluster centers are refined based on the genetic algorithm. The searching capability of genetic algorithms is used in an effective way to find the appropriate cluster centers such that a similarity metric of the resulting clusters is optimized. The selection, crossover, and mutation processes of the genetic algorithm help to refine the cluster centers. It classifies the lung nodules as being benign and malignant based on the lung's size (small or large), shape (spherical or elliptical), and intensity. More information on selection of fitness functions shall be obtained from Veerakumar and Ravichandran [2].

## 3. Results and Discussion

The dataset encompasses 302 lung CT images collected from 23 patients. The dataset was collected privately from cancer diagnostic centers and it comprised nodules of various types and numbers. Other supporting information, including risk factors like age, gender, are also collected and used for feature matrix construction. Totally 815 nodules were accounted in the dataset among which 20% of CT images did not show any nodules. Nodules categories are malignant and benign and were in spherical, elliptical, and irregular shapes. Image acquisition is done by extracting the lung lobes from the provided lung CT image. This step eliminates the portion in the CT image which has patient information embedded. This is done to reduce the false positive produced in the later stages because shape and intensity are considered for nodule detection and classification.

Table 1 shows the evaluation of all 7 algorithms based on recall ratio. The recall ratio of Otsu algorithm is majorly distributed near 1, which indicates that the performance of Otsu is good. The recall value of watershed is spread over a range because of some oversegmentation problems. The distribution of the recall ratio of global region based segmentation indicates that it still involves improvisation. It can be achieved by careful selection of seed points. Most of the recall ratio of Ant Colony Optimization is situated below 0.5 which indicates that much more work has to be done for enhancing the performance.

**Figure 8:**
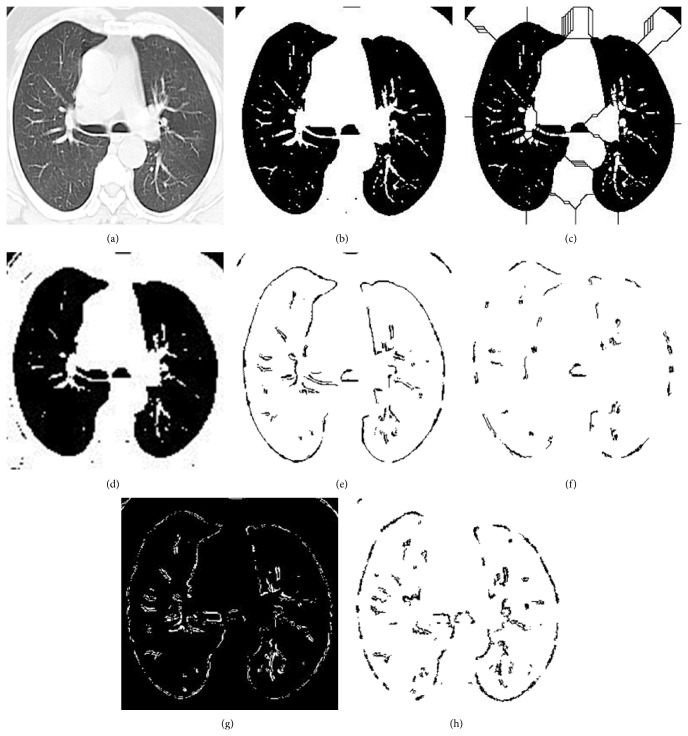
(a) Input lung CT image. (b) Otsu output image. (c) Watershed output image. (d) Global region based segmentation output image. (e) Ant colony optimization output image. (f) Refined ACO output image.  (g) Logical ACO output image. (h) Variant ACO output image.

The improvisation work carried out is evaluated further. Refined ant colony has more room for improvement but gives better performance than the normal ant colony optimization algorithm. It helps in the reduction of noise detection. Logical ant colony optimization visibly shows better performance but still there are wrongly detected nodules. It indicates distribution of recall value above 1. In variant ant colony optimization, though there is distribution of the recall ratio over a range, it shows better performance above all the proposed approaches.  Figure 8(a) is the input given to all algorithms and Figures 8(b)–8(h) are the outputs got from respective algorithms.

Specificity and sensitivity are also used for the evaluation purpose. The average sensitivity and specificity values of all the images are taken and tabulated in Table 2. Specificity measures the proposition of negatives while sensitivity measures the proposition of positives correctly identified. From sensitivity values, we see that Otsu, watershed, and global region based segmentation algorithms are able to detect nodules positively. The sensitivity graphs and specificity tables show a little degradation in the detection of nodules because they include portions which are not actually nodules. The variations in ACO algorithms are better in detection of nodules than the normal ACO algorithm.

Accuracy and precision are also used to evaluate the process. Accuracy measures the degree of closeness of measurement of a quantity to the actual value of the quantity. Precision is the degree to which repeated measurements under unchanged condition show the same result.  Table 3 gives accuracy of all the seven algorithms with their average accuracy value. It depicts the fact that the existing algorithms show a significant accuracy values while, in the proposed algorithms, variant ACO shows higher accuracy than other ACOs.  Table 3 tabulates the average precision of the algorithms. It significantly shows that variant ACO is better in detection of lung nodules than all other algorithms. Logical ACO is not good in detection because we use the output of refined ACO as the input. This reduces precision value further.

**Figure 9:**
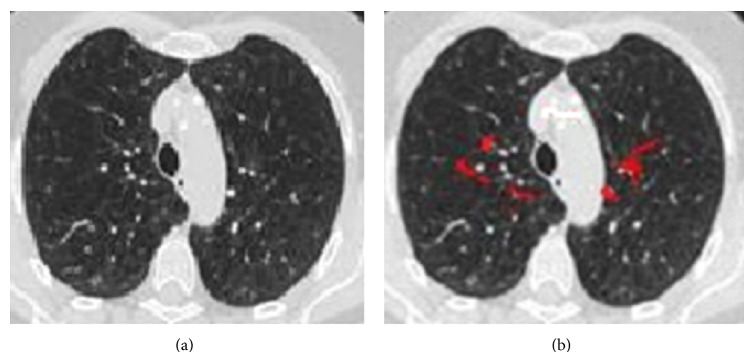
(a) Input image with nodules. (b) Nodule detected output image.

Figure 9(a) shows the input CT image with lung nodules present and Figure 9(b) shows the detected portions of the image as nodules. The evaluation of lung nodule detection is done using the parameters accuracy and precision.

**Figure 10:**
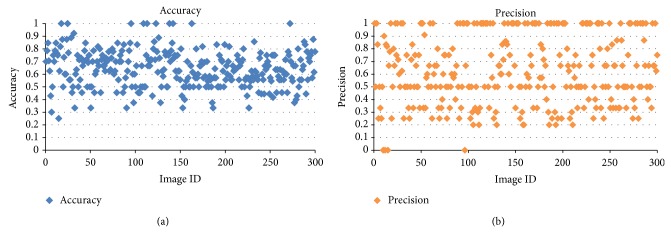
(a) Accuracy for 302 images. (b) Precision for 302 images for hierarchical algorithm based clustering.

**Figure 11:**
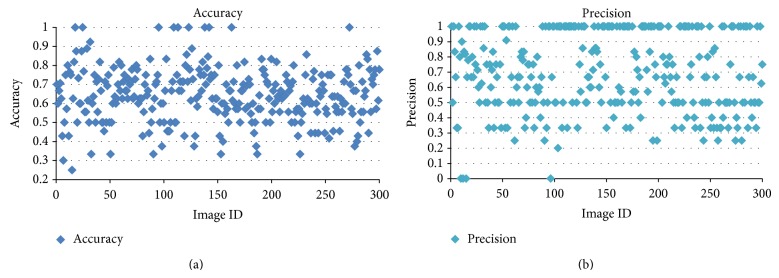
(a) Accuracy for 302 images. (b) Precision for 302 images for genetic algorithm based clustering.

The image dataset has images with and without nodules. The precision and accuracy obtained using hierarchical agglomerative clustering algorithm are 63.7% and 64%, respectively. Figures 10(a) and 10(b) give the graph for accuracy and precision of hierarchical based clustering.  Figure 11(a) gives accuracy for 302 images. For some images (image IDs 18, 25), the proposed algorithm has best clustering accuracy. This is because lung nodules were identified exactly and there were no improper identifications of lung nodules. This gives 100% accuracy for those images. The variation in accuracy is because for some lung images even the lung edges were identified as nodules. It contributes to the false negatives which subsequently reduces the accuracy. The accuracy for the all the images was calculated and the overall accuracy found to be 64%.

Similarly precision value is also calculated for all images and overall precision was obtained. For some images, number of nodules present is zero or very less. For those images, the correctly identified nodules (TP) are very less or even zero. This reduces the precision value which is 0 or close to zero. The same reason holds for precision value 1. For images with no or less nodules, the incorrectly identified nodules were close to zero, thus increasing the precision value close to 1. Also, for those images, there were no incorrectly identified nodules.  Figure 11(b) gives the precision graph for 302 images. Precision for each image is plotted against the image ID. The overall precision obtained for the proposed algorithm is 70%.

This paper discussed a bold and novel attempt in applying ant colony optimization algorithm to pulmonary nodule detection, which is a breakthrough in ACO research in medical imaging. We also suggested various improvements to ACO in three levels: iteration based, logical, and hybrid. The proposed methodology helps to cluster malignant nodules typically based on shape, intensity, and size. Variant Ant Colony Optimization algorithm greatly helps in reducing the number of false positives (FPs). The size and intensity based filtering of nodules helps to reduce the clustering process and the genetic algorithm based clustering identifies the benign and malignant nodules of the lung CT images. False positives still exist because the lung edges were also taken as nodules center in the proposed black circular neighborhood algorithm. To avoid the existing FPs, lung lobe segmentation shall be performed before applying black circular neighborhood algorithm. In addition, variant ACO shall be extended to include the output of other edge detection algorithms.

## Figures and Tables

**Algorithm 1:**
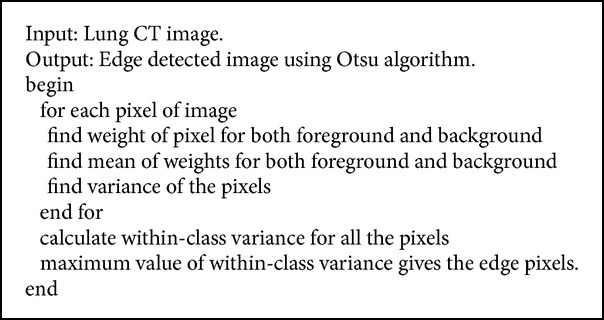
Otsu algorithm.

**Algorithm 2:**
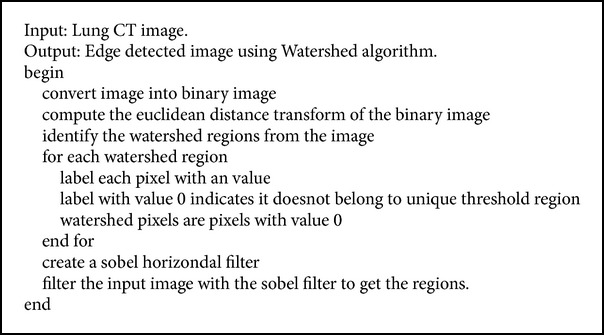
Watershed algorithm.

**Algorithm 3:**
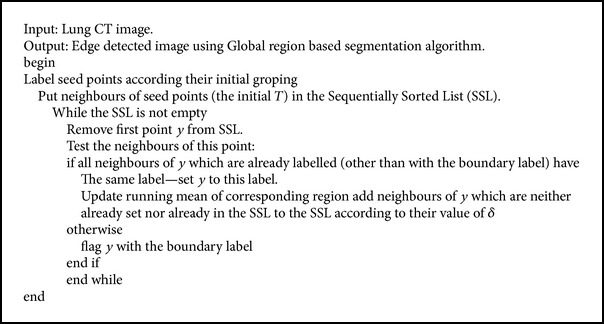
Global region based segmentation algorithm.

**Algorithm 4:**
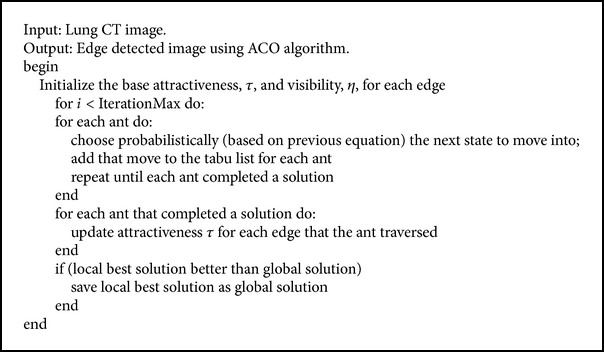
Ant colony optimization algorithm.

**Algorithm 5:**
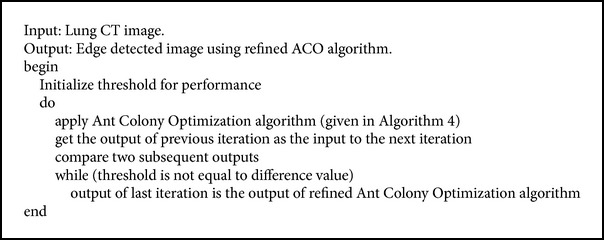
Refined ant colony optimization algorithm.

**Algorithm 6:**
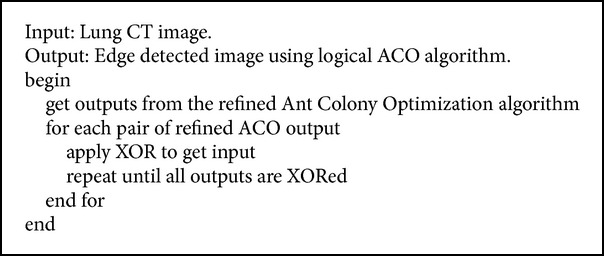
Logical ant colony optimization algorithm.

**Algorithm 7:**
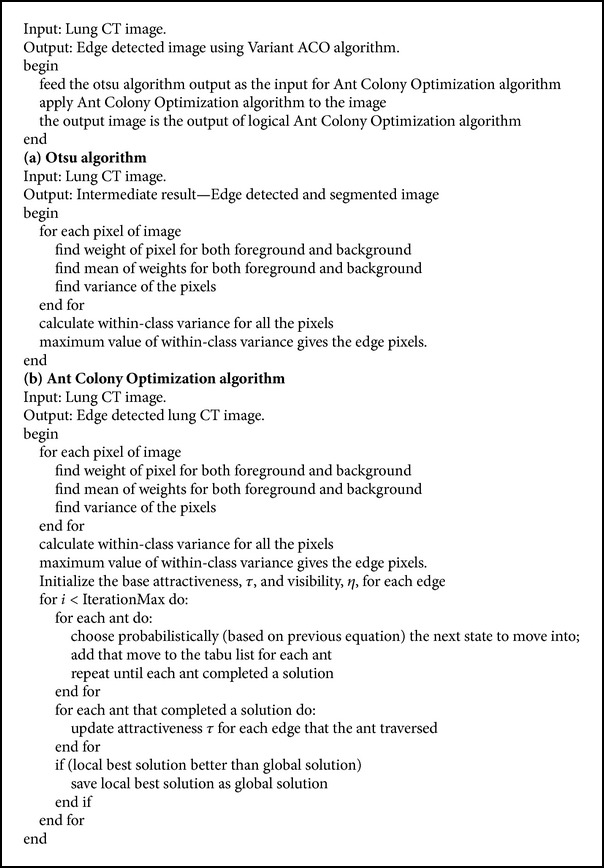
Variant ant colony optimization for edge detection.

**Table 1:** Recall ratio of all seven algorithms for nodule detection.

Algorithm	Otsu	Watershed	Region growing	ACO	Refined ACO	Logical ACO	Variant ACO
Recall ratio	1.50	1.25	1.94	0.69	0.81	0.40	1.07

**Table 2:** Average sensitivity for nodule detection of various algorithms.

Algorithm	Otsu	Watershed	Region growing	ACO	Refined ACO	Logical ACO	Variant ACO
Sensitivity	0.60	0.55	0.60	0.51	0.53	0.53	0.65
Specificity	0.61	0.55	0.64	0.29	0.31	0.16	0.70

**Table 3:** Average accuracy value for nodule detection of all seven algorithms.

Algorithm	Otsu	Watershed	Region growing	ACO	Refined ACO	Logical ACO	Variant ACO
Accuracy	0.60	0.57	0.68	0.43	0.47	0.34	0.77
Precision	0.60	0.47	0.46	0.37	0.36	0.30	0.50
